# Cross-Sectional study on cranial shape measurement values of 2,165 preterm infants in Beijing

**DOI:** 10.3389/fped.2026.1744603

**Published:** 2026-06-10

**Authors:** Rui Li, Miao Yu, Jing Wang, Nuo Ma, Jing Pan, Jinyang Bai, Xinyu Huang, Hua Chen

**Affiliations:** 1 Neonatal Intensive Care Unit, Peking Univeristy Third Hospital, Beijing, China; 2 Department of Intensive Care Unit, Peking Univeristy Third Hospital, Beijing, China; 3 Peking Univeristy School of Nursing, Beijing, China

**Keywords:** cranial shape, Cranial Shape Measurement Values, Cross-sectional study, postural cranial abnormality, preterm infant

## Abstract

**Background:**

The incidence rate of postural cranial abnormality is high. They often accompany facial asymmetry, affecting the appearance and potentially even the development of the nervous system in affected children. To investigate the epidemiological characteristics of the detection rate of postural cranial abnormality in preterm infants, providing a basis for early diagnosis and intervention timing.

**Methods:**

A retrospective study was conducted on cranial measurement data from 2,165 preterm infants who visited the Child Health Care Center of Peking University Third Hospital for the first time between January 1, 2022, and April 30, 2025. Collect basic information of premature infants. Cranial shape data, including Cranial Vault Asymmetry, Cranial Vault Asymmetry Index and Cephalic Ratio, were acquired using the STARscanner 2.0 laser data acquisition system. Preterm infants were divided into three groups based on corrected age: 0–2 months group, 3–4 months group, and 5–6 months group.

**Results:**

This study analyzed 2,165 infants and detected postural cranial abnormality in 1,420 cases. Grouped by corrected age, the postural cranial abnormality was highest in the 0–2 month group (81.2%, 371/457), followed by the 5–6 month group (74.2%, 495/667), and lowest in the 3–4 month group (53.2%, 552/1,041). Upon subdivision of deformation types, it was observed that the detection rate of plagiocephaly fluctuated in a “V” shape among the 0–2, 3–4, and 5–6 month groups (42.2% vs 33.3% vs 37.3%); the detection rate of brachycephaly significantly increased with age (12.7% vs 9.0% vs 19.8%); asymmetric brachycephaly showed no significant change between the 0–2 month and 5–6 month groups (8.8% vs 8.5%); and the detection rate of scaphocephaly significantly decreased between the 0–2 month and 5–6 month groups (17.5% vs 8.5%).

**Conclusion:**

The incidence of postural cranial abnormality is high among Chinese preterm infants, with plagiocephaly being the predominant type. The prevalence of cranial abnormalities shows a U-shaped trend with increasing corrected age (first decreasing then increasing), indicating the necessity for early and continuous intervention. This study provides evidence for incorporating cranial shape screening into routine follow-up for preterm infants and advancing the intervention window to the corrected age of 0–2 months.

## 1 Background

Postural cranial abnormality refers to abnormal head shape in early infancy caused by external forces acting on the skull (1). Unlike craniosynostosis, which involves premature fusion of cranial sutures, postural cranial abnormality results from prolonged external pressure on the malleable infant skull, leading to characteristic deformations without suture fusion clinically classified as plagiocephaly, brachycephaly, and scaphocephaly.

Based on the pattern of cranial deformation, postural cranial abnormality is clinically classified into three main types: plagiocephaly (Plagiocephaly is the most common cranial abnormality in infants. In severe cases of plagiocephaly, there is often asymmetry in the ears, orbits, cheeks, and mandible), brachycephaly (The characteristic of brachycephaly is an abnormal skull length, resulting in an excessively wide head), and scaphocephaly (scaphocephaly is a condition where a child's head is elongated and narrowed, with the anteroposterior diameter of the skull significantly greater than the lateral diameter). Asymmetrical brachycephaly represents a mixed phenotype where features of both plagiocephaly and brachycephaly coexist (2).

Asymmetrical cranial growth is often accompanied by facial asymmetry, affecting appearance (1). This may also cause parental concerns and social reactions (3). Furthermore, studies have shown a significant association between the severity of plagiocephaly and developmental delays (4). Mild to moderate postural cranial abnormality can often be corrected or significantly improved through early positional therapy or physical therapy (5). As infants grow, cranial bone hardness increases, head movement becomes more frequent and less controllable, making correction more difficult and costlier. However, early detection and early treatment can lead to better treatment outcomes (6). Therefore, the early identification and timely intervention of abnormal cranial shapes in premature infants have become a pressing issue that needs to be addressed in clinical practice.

The anomaly detection rate of postural cranial abnormality correlates with infant age. Plagiocephaly is the most common type of postural cranial abnormality in infants. Van Vlimmeren et al. assessed healthy term neonates at birth and 7 weeks using plagiocephalometry, finding the anomaly detection rate increased from 6.1% to 22.1% (7). Currently, research on postural cranial abnormality in preterm infants is limited. Preterm infants are considered a high-risk group for postural cranial abnormality (8), primarily due to the plasticity of the premature infant's skull (1), as well as cesarean section and the compression caused by mechanical ventilation (use of mechanical ventilation) factors (9). Postural cranial abnormality affects the appearance of preterm infants and may even threaten their neurodevelopment. However, due to their early birth, preterm infants have a longer potential correction period than term infants if postural cranial abnormality is detected early. A study including 103 preterm infants showed that 99 (96.1%) had postural cranial abnormality (10). In thisstudy, a high percentage of infants suffer from plagiocephaly (skewed head shape). Mild plagiocephaly is the most common. Regardless of the degree of premature birth, a lower percentage of infants exhibit brachycephaly (short head shape) and scaphocephaly (long head shape). An analysis of 530 preterm infants in Chongqing, southern China, using manual measurement based on the Wilbrand standardized protocol, found that plagiocephaly anomaly detection rate decreased while brachycephaly anomaly detection rate increased with increasing corrected age (11). However, international standards may not be applicable to Chinese preterm infants due to racial and gestational age differences.

Previous studies lack consensus on subjective or objective assessment criteria for postural cranial abnormality, and existing standards are mostly for healthy term infants. Epidemiological research on this high-risk preterm population is scarce. With advances in medical technology improving survival rates for very/extremely preterm infants, updated epidemiological data is urgently needed to support clinical practice. This study utilizes more precise 3D scanning technology for objective assessment of postural cranial abnormality. It aims to investigate the epidemiological characteristics of postural cranial abnormality in preterm infants attending a tertiary hospital's child health center in Beijing, China, and to preliminarily establish reference diagnostic criteria for this population, thereby informing early prevention and correction strategies.

## 2 Materials and methods

### 2.1 Study participants

This Cross-sectional study enrolled preterm infants who were registered for follow-up at the Child Health Development Center of our hospital between January 1, 2022, and April 30, 2025. Inclusion criteria: (1) Preterm infants (gestational age at birth <37 weeks); (2) Registered for follow-up at the center with complete medical records. Exclusion criteria: (1) Infants with cranial abnormalities due to craniosynostosis, osteogenesis imperfecta, infantile hydrocephalus, or congenital cervical deformities; (2) Infants with congenital malformations (e.g., gastrointestinal malformations, cyanotic congenital heart disease); (3) Infants who developed severe illnesses affecting growth and development during follow-up; (4) Infants receiving cranial orthotic treatment at the first visit or during follow-up. Excluding infants receiving cranial orthotic treatment was intended to obtain natural cranial morphology data without intervention. In practice, only 12 cases (0.55%) met this criterion, and they were evenly distributed across age groups; sensitivity analysis showed that the exclusion did not affect the main conclusions.

This study has been approved by the Medical Scientific Research Ethics Committee of Peking University Third Hospital (approval number: IRB00006761-M20250752).

### 2.2 Measurement methods and diagnostic criteria for cranial abnormalities

**Figure 1:**
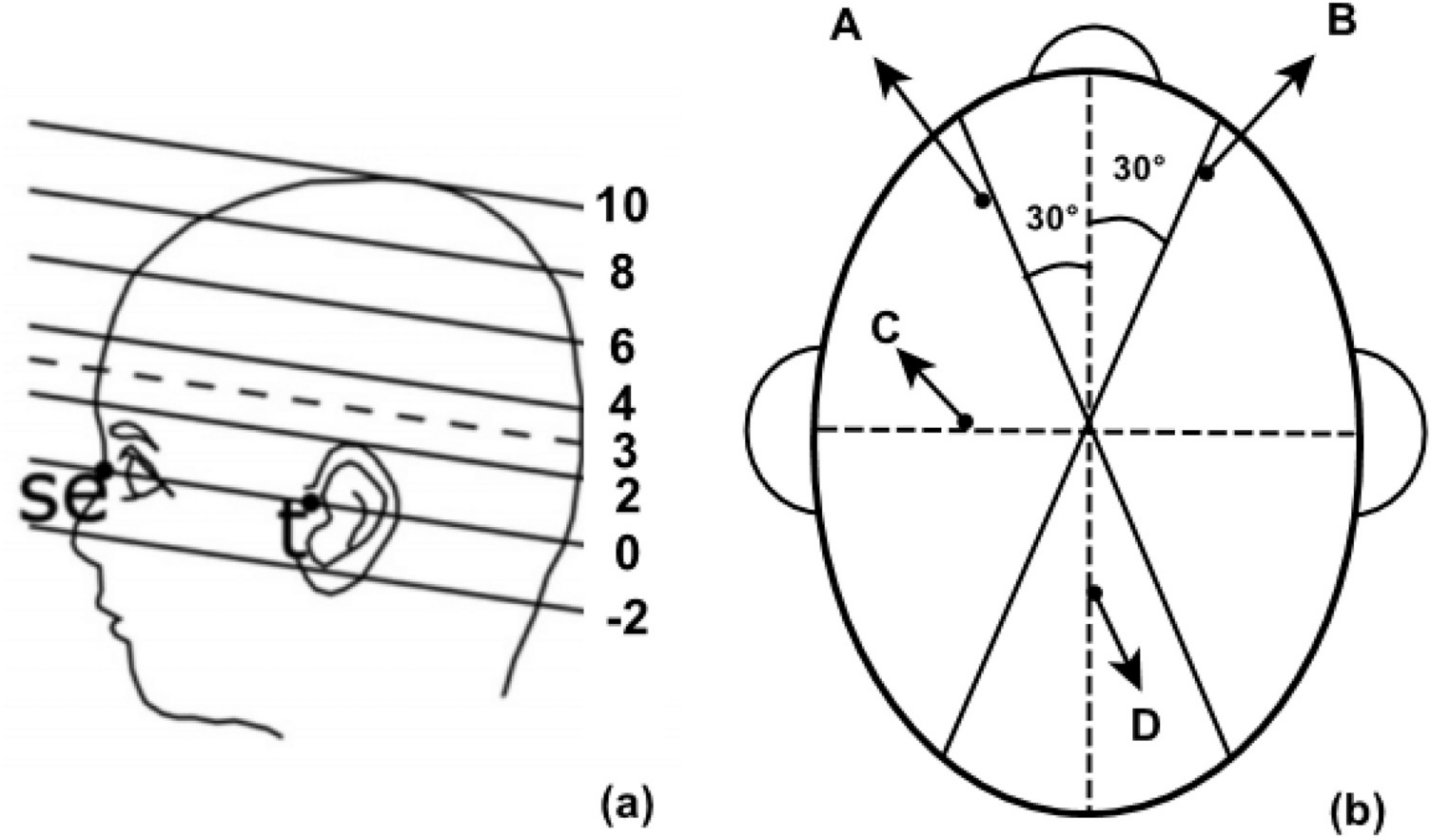
Cranial scan diagrams. **(a)**, Schematic of Level 3 plane; **(b)** Schematic of measurement indices.

Cranial shape was scanned using the STARscanner 2.0 laser data acquisition system. Three-dimensional images were captured using dedicated cranial data analysis software. The software defined the horizontal plane (Level 0) as passing through the glabella (midpoint between eyes) and both tragus points. The region from Level 0 to the vertex was divided into 10 equal segments from bottom to top. All parameters in this study were obtained from the Level 3 cross-section (12) (Figure 1a).

On the Level 3 cross-section, the vertical distances anterior and posterior to the points located 30° left and right of the midline were recorded as A and B (A > B). Cranial Vault Asymmetry (CVA) = A − B. Cranial Vault Asymmetry Index (CVAI) = (A − B)/A*100%. The width and length along the midline of the Level 3 cross-section were recorded as C and D. Cephalic Ratio (CR) = C/D (Figure 1b) (2).

Based on international diagnostic criteria, previous literature, and clinical experience, the diagnostic criteria for the four types of cranial abnormalities in this study were:

Plagiocephaly: Uneven pressure on both sides of the head causing oblique flattening, leading to increased diagonal difference. Abnormal value defined as “CVA ≥ 6 mm or CVAI ≥3.5%” (13).

Brachycephaly: Abnormal cranial length resulting in excessive width and an increased head width/length ratio. Abnormal value defined as “CR  ≥ 0.90” (14).

Scaphocephaly: Significantly increased anteroposterior diameter relative to transverse diameter, resulting in a long, narrow head shape. Abnormal value defined as “CR < 0.75” (15).

Asymmetrical Brachycephaly: Co-occurrence of brachycephaly and plagiocephaly. Abnormal value defined as “CR ≥ 0.90 and simultaneously CVA ≥ 6 mm or CVAI ≥ 3.5%”.

The distinction between normal cases and mild/subclinical cranial shape abnormalities is based on the objective rule that “any of the three-dimensional measurements exceeds the normal reference range but has not reached the diagnostic criteria for confirmed deformity,” and is further validated by clinical palpation. This classification system is directly derived from the Expert Consensus on the Prevention and Management of Positional Cranial Shape Abnormalities in Premature Infants (2026), with quantified refinements, providing good reproducibility and clinical operability.

### 2.3 Statistical analysis

Statistical analysis was performed using SPSS 22.0 software. Measurement data are presented as mean ± standard deviation ($\bar{x}\pm \mathrm{SD}$), and differences between groups were compared using independent samples *t*-tests. Count data are presented as number and percentage (*n*, %), and differences between groups were compared using chi-square (*χ*^2^) tests. A *P*-value < 0.05 was considered statistically significant.

## 3 Results

### 3.1 Basic characteristics of preterm infants

This study included a total of 2,165 preterm infants. Among them, there were 1,175 males (54.3%) and 990 females (45.7%). The gestational age at birth ranged from 23.1 to 37.0 weeks, with a mean of (33.56 ± 2.65) weeks. Grouped by gestational age at birth, there were 113 cases (5.2%) of extremely preterm infants (<28 weeks), 374 cases (17.3%) of preterm infants (28–31 weeks), and 1,678 cases (77.5%) of late preterm infants (32–36 weeks). Grouped by corrected age at the time of consultation, there were 457 cases (21.1%) in the 0–2 months group, 1,041 cases (48.1%) in the 3–4 months group, and 667 cases (30.8%) in the 5–6 months group. There were no statistically significant differences in gestational age at birth (*F* = 0.532, *P* = 0.587) or gender composition (*χ*^2^ = 0.407, *P* = 0.816) among the different corrected age groups, indicating balanced baseline characteristics and comparability across groups. See Table 1 for details.

**Table 1:** Basic characteristics of preterm infants (*n* = 2,165).

Group	Cases (*n*, %)	Gestational Age [weeks, (x¯ ± SD)]	Gender	
			Male (*n*, %)	Female (*n*, %)
Corrected 0–2m	457 (21.1)	33.7 ± 2.7	242 (53.0)	215 (47.0)
Corrected 3–4m	1,041 (48.1)	33.6 ± 2.8	569 (54.7)	472 (45.3)
Corrected 5–6m	667 (30.8)	33.5 ± 2.8	364 (54.6)	303 (45.4)
F/*χ*^2^		0.532	0.407	
*P*		0.587	0.816	

### 3.2 Detection of different types of postural cranial abnormality

Among the 2,165 preterm infants, 1,420 cases (65.6%) presented with cranial deformities. This study employed a non-mutually exclusive classification method for statistical analysis of cranial deformities, meaning that a single infant could meet the diagnostic criteria for multiple deformities simultaneously. The specific diagnostic categories and their overlapping relationships are as follows:

(1) Plagiocephaly (CVA ≥ 6 mm or CVAI ≥ 3.5%): 789 cases (36.4%); (2) Brachycephaly (CR ≥ 0.90): 284 cases (13.1%); (3) Asymmetrical brachycephaly: Defined as cases meeting the diagnostic criteria for both plagiocephaly and brachycephaly simultaneously. That is, asymmetrical brachycephaly is the intersection of plagiocephaly and brachycephaly, not an independent category (16). Verification showed that the number of cases simultaneously meeting plagiocephaly = 1 and brachycephaly = 1 was 142 (6.6%), which is completely consistent with the asymmetrical brachycephaly coding. Cases of asymmetrical brachycephaly are also counted in the total numbers for both plagiocephaly and brachycephaly; (4) Scaphocephaly (CR < 0.75): 303 cases (14.0%).

Overlaps between deformity categories: There were 142 cases of overlap between plagiocephaly and brachycephaly (i.e., asymmetrical brachycephaly); there were 8 cases (0.4%) of overlap between plagiocephaly and scaphocephaly, meaning these infants simultaneously met the plagiocephaly criteria (CVA ≥ 6 mm or CVAI ≥ 3.5%) and the scaphocephaly criteria (CR < 0.75); there was no overlap (0 cases) between brachycephaly and scaphocephaly, as the diagnostic criteria of CR ≥ 0.90 and CR < 0.75 are mutually exclusive. The union of these three main deformity types (plagiocephaly, brachycephaly, scaphocephaly) was 1,226 cases. Additionally, 194 cases (9.0%) were classified as mild/subclinical cranial shape abnormalities. According to the Expert Consensus on the Prevention and Management of Positional Cranial Shape Abnormalities in Premature Infants (2026), cranial shape abnormalities are classified into three degrees of severity. The present study used the following quantitative thresholds for classification, as supplemented in Table 2.

**Table 2:** Grading diagnostic indicators for cranial deformities (plagiocephaly, brachycephaly, scaphocephaly).

**Classification**	**Plagiocephaly-related indicators**	**Brachycephaly-related indicators**	**Scaphocephaly-related indicators**	**Clinical significance**
normal	CVA < 4 mm and CVAI < .5%	CR 0.75–0.90	—	no intervention required
mild/subclinical abnormalities	CVA 4–5.9 mm or CVAI 2.5–3.49%	CR 0.91–0.95	CR 0.70–0.74	positional management, regular follow-up
confirmed deformities	CVA ≥ 6 mm and CVAI ≥ .5%	CR ≥ 0.90	CR < 0.75	active intervention

**Figure 2:**
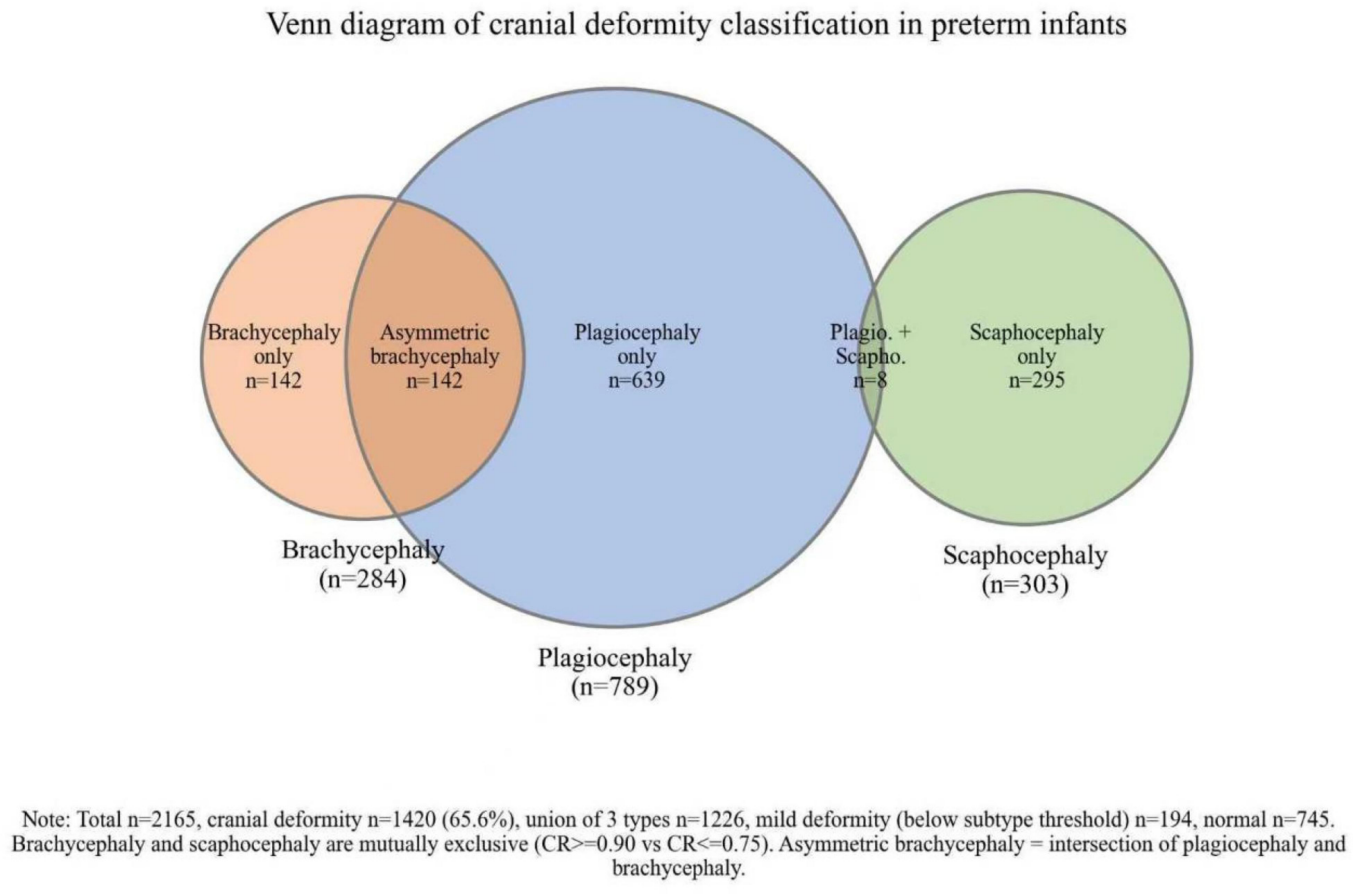
Venn diagram for classification of abnormal cranial shapes in premature infants.

Regarding the potential addition of an “Asymmetrical Scaphocephaly” category (i.e., plagiocephaly combined with scaphocephaly): In this study, there were only 8 overlapping cases of plagiocephaly and scaphocephaly (0.4% of the total sample). The sample size was too small to have statistical significance for separate analysis; therefore, it was not established as an independent category. The mutually exclusive classification statistics for each deformity subtype are as follows: Normal: 745 cases (34.4%), Plagiocephaly only: 639 cases (29.5%), Brachycephaly only: 142 cases (6.6%), Scaphocephaly only: 295 cases (13.6%), Asymmetrical brachycephaly (Plagiocephaly + Brachycephaly): 142 cases (6.6%), Plagiocephaly + Scaphocephaly: 8 cases (0.4%), Mild deformity: 194 cases (9.0%) as shown in Figure 2.

### 3.3 Detection of postural cranial abnormality in different correction gestational age groups

Based on the grouping by the corrected age group, it was found that the proportion of postural cranial abnormality in the group with a corrected age of 0–2 months was the highest, at 81.2% (371/457), followed by the group with a corrected age of 5–6 months (74.2%, 495/667), and the group with a corrected age of 3-4 months had the lowest proportion of postural cranial abnormality (53.2%, 554/1,041). The differences in the distribution of postural cranial abnormality among different groups were statistically significant (*χ*^2^ = 141.796, *P* < 0.001).

Further pairwise comparisons showed that in plagiocephaly, the detection rate in the group with a corrected age of 0–2 months (42.2%) was significantly higher than that in the group with a corrected age of 3–4 months (33.3%) (*χ*^2^ = 10.908, *P* = 0.001), while there was no statistically significant difference between the group with a corrected age of 5–6 months (37.3%) and the group with a corrected age of 0–2 months (*P* = 0.099). In brachycephaly, the detection rate in the group with a corrected age of 5–6 months (19.8%) was significantly higher than that in the group with a corrected age of 0–2 months (12.7%) (*χ*^2^ = 9.729, *P* = 0.002) and the group with a corrected age of 3–4 months (9.0%) (*χ*^2^ = 40.998, *P* < 0.001). In asymmetrical brachycephaly, the detection rate in the group with a corrected age of 3–4 months (4.3%) was significantly lower than that in the group with a corrected age of 0–2 months (8.8%) (*χ*^2^ = 11.644, *P* = 0.001), and there was no statistically significant difference between the group with a corrected age of 5–6 months (8.5%) and the group with a corrected age of 0–2 months (*P* = 0.903). In Scaphocephaly, the detection rate in the group with a corrected age of 5–6 months (8.5%) was significantly lower than that in the group with a corrected age of 0–2 months (17.5%) (*χ*^2^ = 20.341, *P* < 0.001) and the group with a corrected age of 3–4 months (15.9%) (*χ*^2^ = 19.613, *P* < 0.001), and there was no statistically significant difference between the group with a corrected age of 0–2 months and the group with a corrected age of 3–4 months (*P* = 0.453). Detailed results are shown in Table 3.

**Table 3:** Detection of postural cranial abnormality by corrected age group (*n* = 2,165).

Group	Total Cases	postural cranial abnormality (*n*, %)	Abnormal Type (*n*, %)			
			Plagiocephaly (*n*, %)	Brachycephaly (*n*, %)	Asymmetrical Brachycephaly (*n*, %)	Scaphocephaly (*n*, %)
Corrected 0–2 m	457	371 (81.2)	193 (42.2)	58 (12.7)	40 (8.8)	80 (17.5)
Corrected 3–4 m	1,041	554 (53.2)	347 (33.3)	94 (9.0)	45 (4.3)	166 (15.9)
*χ* ^2^ ^a^		106.449	10.908	4.670	11.644	0.563
*P*		<0.001	<0.001	0.031	<0.001	0.453
Corrected 5–6 m	667	495 (74.2)	249 (37.3)	132 (19.8)	57 (8.5)	57 (8.5)
*χ* ^2^ ^b^		7.447	2.730	9.729	0.015	20.341
*P*		0.006	0.099	0.002	0.903	<0.001
*χ* ^2^ ^c^		76.923	2.860	40.998	12.911	19.613
*P*		<0.001	0.094	<0.001	<0.001	<0.001
Total	2,165	1,420 (65.5)	789 (36.4)	284 (13.1)	142 (6.6)	303 (14.0)

a Comparison between Corrected 3–4 m group and Corrected 0–2 m group.

b Comparison between Corrected 5–6 m group and Corrected 0–2 m group.

c Comparison between Corrected 5–6 m group and Corrected 3–4 m group.

### 3.4 Distribution of Cva, Cvai, and Cr values in preterm infants

**Figure 3:**
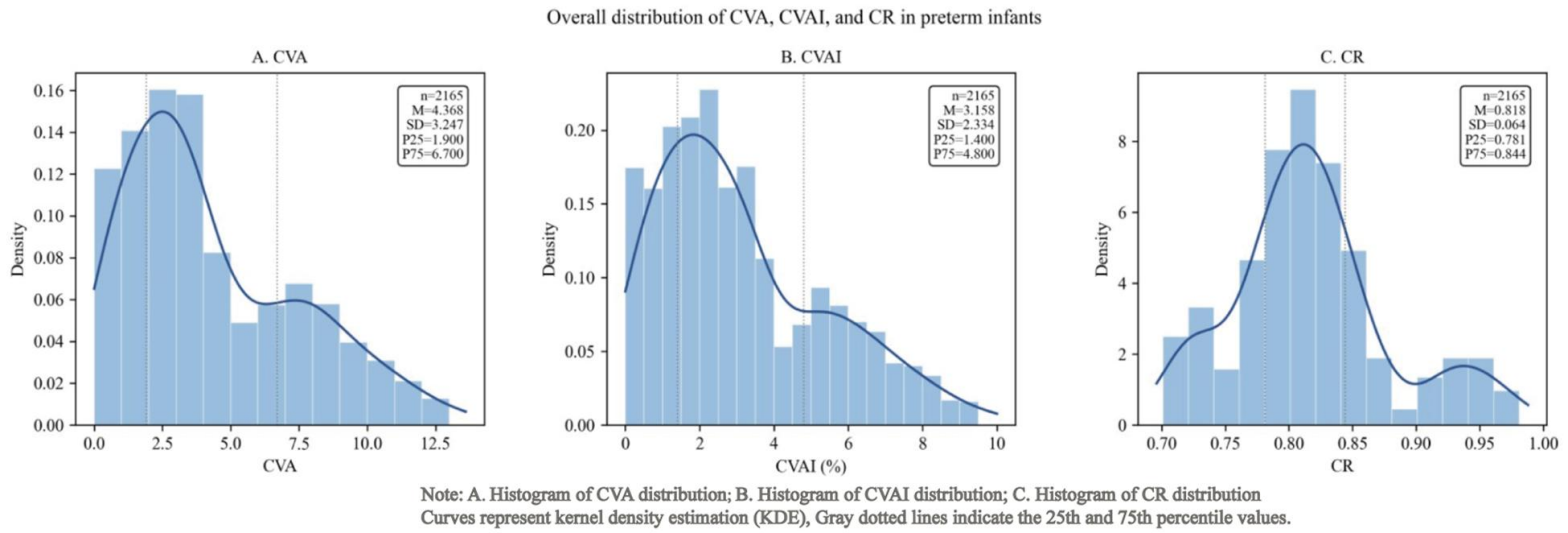
Histogram of the overall distribution of CVA, CVAI and CR in premature infants. Note: **(A)** Histogram of CVA distribution; **(B)** Histogram of CVAI distribution; **(C)** Histogram of CR distribution. Curves represent kernel density estimation (KDE), Gray dotted lines indicate the 25th and 7$th percentile values.

**Figure 4:**
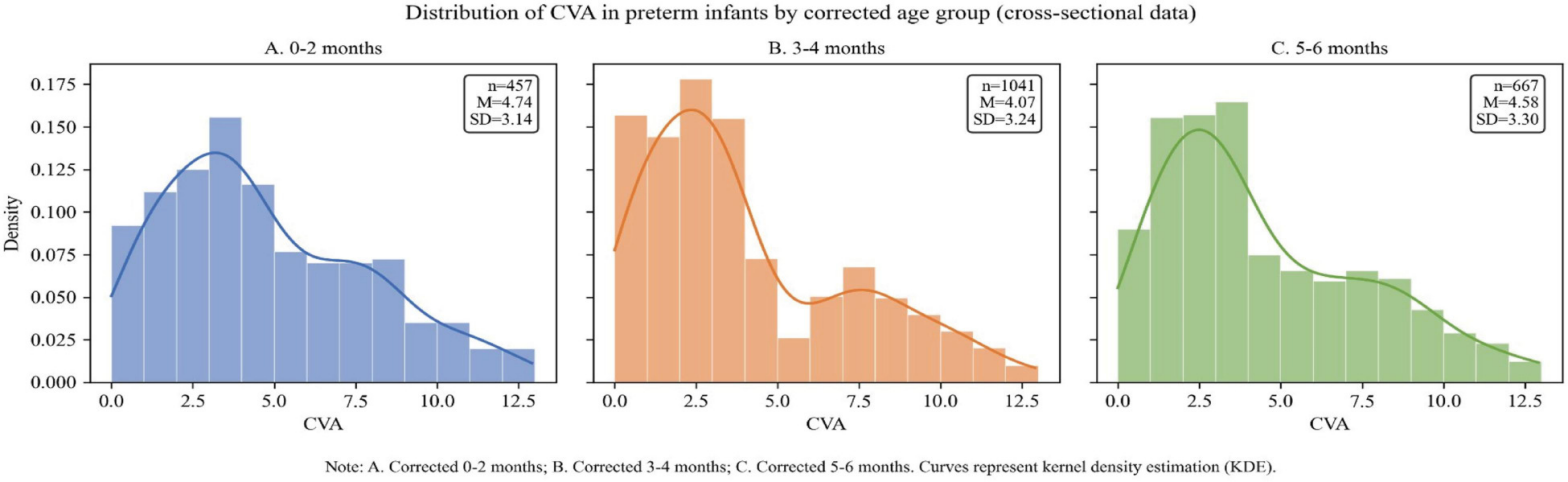
Histogram of CVA distribution in preterm infants of different correction age groups (cross-sectional data). Note: **(A)** Corrected 0-2 months; **(B)** Corrected 3-4 months; **(C)** Corrected 5-6 months. Curves represent kernel density estimation (KDE).

**Figure 5:**
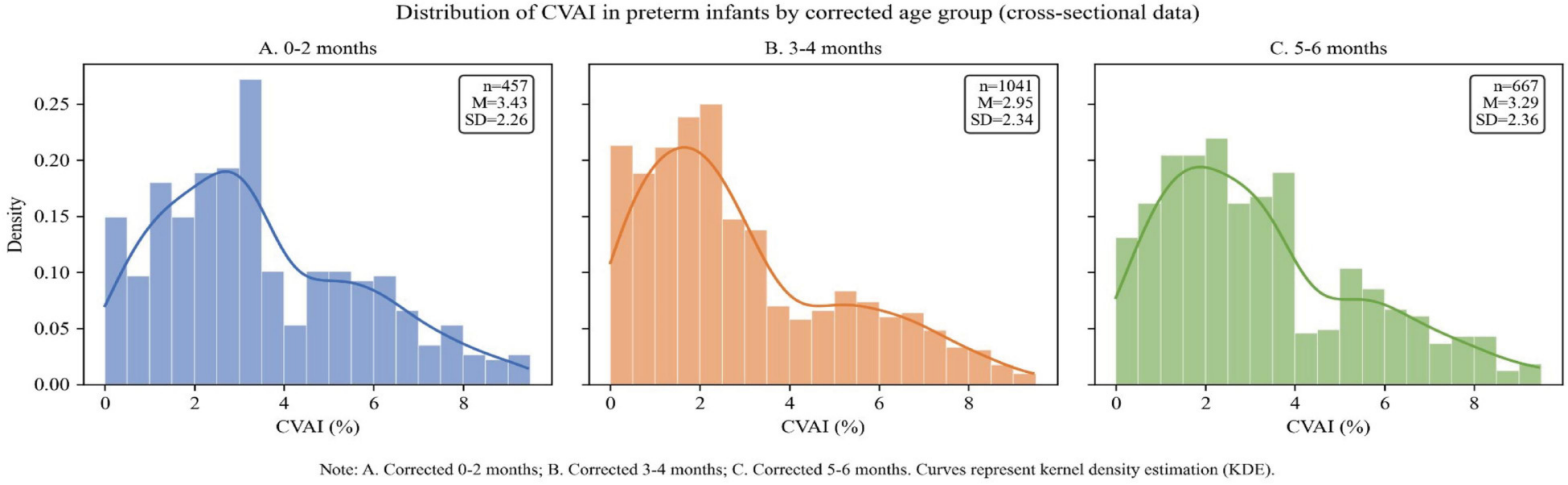
Histogram of CVAI distribution in preterm infants of different correction age groups (cross-sectional data). Note: **(A)** Corrected 0-2 months; **(B)** Corrected 3-4 months; **(C)** Corected 5-6 months. Curves represent kemel density estimation (KDE).

**Figure 6:**
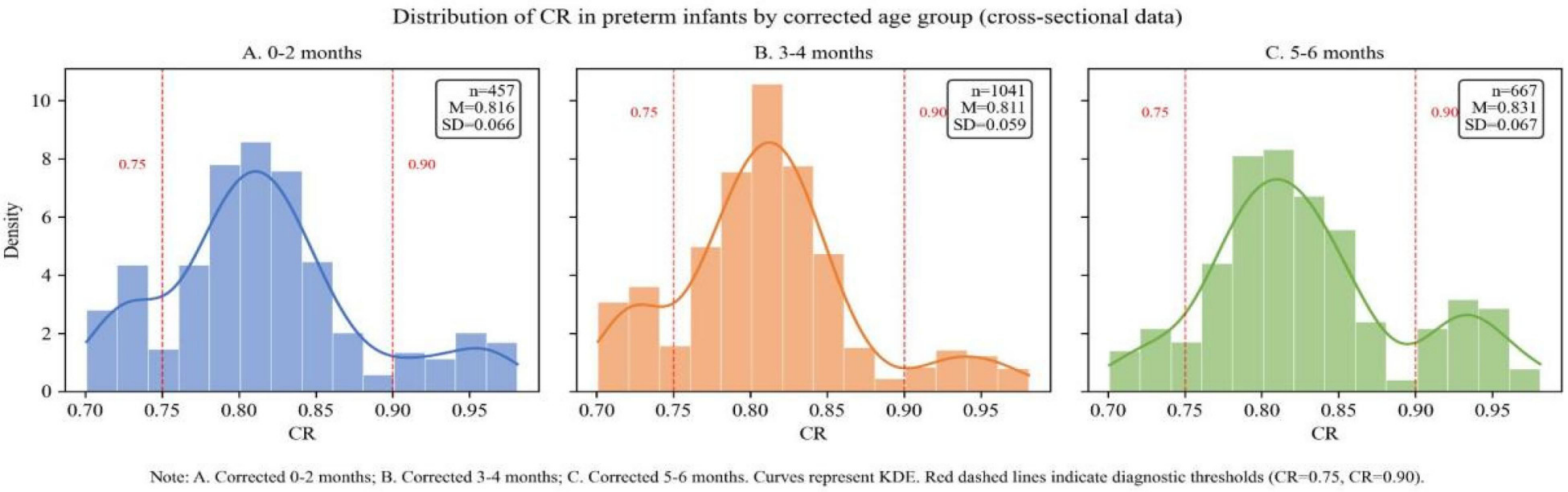
Histogram of CR distribution in preterm infants of different correction age groups (cross-sectional data).

As shown in Figures 3–6, the 25th percentile (*P*₂₅) and 75th percentile (*P*₇₅) for CVA in preterm infants were 1.70 and 6.30, respectively. For CVAI, *P*_25_ and *P*₇₅ were 1.20 and 4.50, respectively. For CR, P_25_ and P_75_ were 0.77 and 0.86, respectively. Details are shown in Table 4. Mean ± standard deviation of CR by gender, males were 0.819 ± 0.064 and females were 0.817 ± 0.064(*t* = 0.846, *P* = 0.398). For CVA, males were 4.488 ± 3.310 and females were 4.226 ± 3.167 (*t* = 1.873, *P* = 0.061). For CVAI, males were 3.207 ± 2.371 and females were3.100 ± 2.289 (*t* = 1.061, *P* = 0.289).

**Table 4:** Percentile values for CVA, cVAI, and CR.

Percentile	CVA	CVAI	CR
3	0.20	0.10	0.713
10	0.80	0.50	0.734
25	1.90	1.40	0.781
50	3.40	2.60	0.813
75	6.75	4.80	0.844
90	9.30	6.70	0.923
97	11.40	8.30	0.961

### 3.5 Detection of postural cranial abnormality by gender

Within each age group, there were no significant differences in the incidence rates of plagiocephaly, brachycephaly, asymmetrical brachycephaly, or scaphocephaly between males and females (all *P* > 0.05, Table 5).

**Table 5:** Detection of postural cranial abnormality by gender (*n* = 2,165).

Type	Corrected 0–2 m Group (*n*, %)				Corrected 3–4 m Group (*n*, %)				Corrected 5–6 m Group (*n*, %)			
	**Male**	**Female**	***χ*²**	*P*	**Male**	**Female**	**χ²**	*P*	**Male**	**Female**	**χ²**	*P*
Plagiocephaly	109 (45.0)	84 (39.1)	1.664	0.197	185 (32.5)	162 (34.3)	0.380	0.538	143 (39.3)	106 (35.0)	1.308	0.253
Brachycephaly	36 (14.9)	22 (10.2)	2.215	0.137	56 (9.8)	38 (8.1)	1.007	0.316	69 (19.0)	63 (20.8)	0.351	0.553
Asymmetrical Brachycephaly	24 (9.9)	16 (7.4)	0.874	0.350	26 (4.6)	19 (4.0)	0.185	0.667	29 (8.0)	28 (9.2)	0.343	0.558
Scaphocephaly	36 (14.9)	44 (20.5)	2.463	0.117	90 (15.8)	76 (16.1)	0.016	0.901	29 (8.0)	28(9.2)	0.343	0.558

### 3.6 Correlation analysis of postural cranial abnormality with birth gestational age

The preterm infants were divided into three groups according to birth gestational age: <28 weeks group (113 cases), 28–31 weeks group (374 cases), and 32–36 weeks group (1,678 cases). The differences in cranial shape indicators among the three groups were compared. Since the data of all three groups did not conform to the normal distribution (Shapiro–Wilk test, all *P* < 0.05), the Kruskal–Wallis *H*-test was used for group comparison.

The results showed that there was a statistically significant difference in CR values among the three groups (*H* = 8.016, *P* = 0.018). The CR value of the <28 weeks group (0.831 ± 0.065) was higher than that of the 28–31 weeks group (0.824 ± 0.064) and the 32–36 weeks group (0.816 ± 0.064). Pairwise comparisons showed that there was a statistically significant difference between the <28 weeks group and the 32–36 weeks group (*U* = 82,748, *P* = 0.023), and the differences between the other two pairs of comparisons were not statistically significant. There was a statistically significant difference in CVA values among the three groups (*H* = 6.204, *P* = 0.045). Pairwise comparisons showed that the CVA value of the 28–31 weeks group (4.626 ± 3.169) was higher than that of the 32–36 weeks group (4.294 ± 3.260) (*U* = 290,033, *P* = 0.022), and there was no statistically significant difference between the <28 weeks group and the other two groups. There was a statistically significant difference in CVAI values among the three groups (*H* = 8.525, *P* = 0.014). Pairwise comparisons showed that the CVAI value of the 28–31 weeks group (3.372 ± 2.336) was higher than that of the 32–36 weeks group (3.087 ± 2.327) (*U* = 289,045, *P* = 0.017), and the difference between the <28 weeks group and the 32–36 weeks group was close to the significant level (*P* = 0.054). The overall trend indicated that the smaller the birth gestational age, the higher the cranial shape morphological indicator values, suggesting that the degree of postural cranial abnormality was more severe (Table 6).

**Table 6:** Comparison of CR, CVA, and cVAI among different birth gestational age groups.

Indicators	<28 weeks (*n* = 113)	28–31 weeks (*n* = 374)	32–36 weeks(*n* = 1,678)	*H*	*P*
CR	0.831 ± 0.065	0.824 ± 0.064	0.816 ± 0.064	8.016	0.018
CVA	4.620 ± 3.289	4.626 ± 3.169	4.294 ± 3.260	6.204	0.045
CVAI	3.500 ± 2.391	3.372 ± 2.336	3.087 ± 2.327	8.525	0.014

Note: The data are presented as mean ± standard deviation. H represents the Kruskal–Wallis test statistic. Pairwise comparisons were conducted using the Mann–Whitney *U*-test, with Bonferroni correction for the significance level alpha = 0.0167.

### 3.7 Association analysis between gestational age at birth and postural cranial abnormality

The overall analysis without grouping by corrected gestational age showed that there were statistically significant differences in the detection rates of plagiocephaly (χ^2^ = 11.741, *P* = 0.003), brachycephaly (χ^2^ = 6.247, *P* = 0.044), Scaphocephaly (χ^2^ = 9.886, *P* = 0.007), and postural cranial abnormality (χ^2^ = 10.481, *P* = 0.005) with respect to gestational age at birth. The smaller the gestational age at birth, the higher the detection rates of plagiocephaly (47.8% vs 40.9% vs 34.7%), brachycephaly (17.7% vs 16.0% vs 12.2%), and postural cranial abnormality (73.5% vs 71.1% vs 63.8%). The detection rate of Scaphocephaly increased with the increase in gestational age (7.1% vs 10.7% vs 15.2%). There was no statistically significant difference in asymmetrical brachycephaly with gestational age (χ^2^ = 2.904, *P* = 0.234). See Table 7 for details.

**Table 7:** Detection of postural cranial abnormality in different birth gestational Age groups (*n* = 2,165).

type	<28 weeks (*n* = 113)	28th to 31st week (*n* = 374)	32–36 weeks(*n* = 1,678)	chi2	*P*
Plagiocephaly	54 (47.8)	153 (40.9)	582 (34.7)	11.741	0.003
Brachycephaly	20 (17.7)	60 (16.0)	204 (12.2)	6.247	0.044
Asymmetrical Brachycephaly	10 (8.8)	30 (8.0)	102 (6.1)	2.904	0.234
Scaphocephaly	8 (7.1)	40 (10.7)	255 (15.2)	9.886	0.007
postural cranial abnormality	83 (73.5)	266 (71.1)	1,071 (63.8)	10.481	0.005

Further stratified analysis was conducted within each correction age group based on birth gestational age (Table 8). Due to the relatively small sample size of the <28 weeks group in each correction age group (25–46 cases), the differences in gestational age stratification among the various deformation types within each group did not reach statistical significance (all *P* > 0.05). In the correction 0–2 months age group, the detection rate of plagiocephaly in <28 weeks premature infants (52.4%) was higher than that in the 32–36 weeks group (39.7%), but the difference did not reach statistical significance (χ^2^ = 2.836, *P* = 0.242); in the correction 3–4 months age group, the detection rate of plagiocephaly in <28 weeks premature infants (44.0%) was higher than that in the 32–36 weeks group (32.6%), and the difference also did not reach statistical significance (χ^2^ = 2.140, *P* = 0.343). These results suggest that the influence of birth gestational age on postural cranial abnormality has statistical significance at the overall level, but in the subgroup analysis within each correction age group, it was limited by the insufficient sample size of the <28 weeks group and failed to detect significant differences.

**Table 8:** Detection of postural cranial abnormality in different birth size groups within each correction Age group.

Group/type	**<28 weeks**	**28th to 31st week**	**32**–**36 weeks**	**chi2**	***P***
Corrected 0–2 m Group					
Plagiocephaly	22/42 (52.4%)	55/123 (44.7%)	116/292 (39.7%)	2.836	0.242
Brachycephaly	6/42 (14.3%)	17/123 (13.8%)	35/292 (12.0%)	0.369	0.832
Asymmetrical Brachycephaly	4/42 (9.5%)	11/123 (8.9%)	25/292 (8.6%)	0.050	0.975
Scaphocephaly	4/42 (9.5%)	18/123 (14.6%)	58/292 (19.9%)	3.679	0.159
postural cranial abnormality	35/42 (83.3%)	100/123 (81.3%)	236/292 (80.8%)	0.153	0.926
Corrected 3–4 m Group					
Plagiocephaly	11/25 (44.0%)	34/91 (37.4%)	302/925 (32.6%)	2.140	0.343
Brachycephaly	3/25 (12.0%)	9/91 (9.9%)	82/925 (8.9%)	0.381	0.826
Asymmetrical Brachycephaly	1/25 (4.0%)	4/91 (4.4%)	40/925 (4.3%)	0.007	0.996
Scaphocephaly	2/25 (8.0%)	11/91 (12.1%)	153/925 (16.5%)	2.432	0.296
postural cranial abnormality	13/25 (52.0%)	47/91 (51.6%)	494/925 (53.4%)	0.118	0.943
Corrected 5–6 m Group					
Plagiocephaly	21/46 (45.7%)	64/160 (40.0%)	164/461 (35.6%)	2.456	0.293
Brachycephaly	11/46 (23.9%)	34/160 (21.2%)	87/461 (18.9%)	0.952	0.621
Asymmetrical Brachycephaly	5/46 (10.9%)	15/160 (9.4%)	37/461 (8.0%)	0.618	0.734
Scaphocephaly	2/46 (4.3%)	11/160 (6.9%)	44/461 (9.5%)	2.197	0.333
postural cranial abnormality	35/46 (76.1%)	119/160 (74.4%)	341/461 (74.0%)	0.101	0.951

## 4 Discussion

This study is the first to systematically elucidate the epidemiological characteristics of postural cranial abnormality in preterm infants in Beijing, China. Based on a large-sample cross-sectional analysis of 2,165 preterm infants, the overall detection rate of postural cranial abnormality was found to be high at 65.5%. Plagiocephaly (55.6%) was the predominant type, with a significantly higher detection rate than scaphocephaly (21.4%), brachycephaly (20.0%), and asymmetrical brachycephaly (10.0%). Furthermore, the detection rate showed a U-shaped trend with increasing corrected age.

The overall detection rate of 65.5% in this study is lower than the 89.2% reported in a study of 530 preterm infants in Chongqing, China (2019) (11), but higher than rates reported in smaller Japanese (*n* = 94, 41.5%) and German (*n* = 56, 34.0%) studies (9, 17). This discrepancy may be related to factors such as measurement techniques, and diagnostic criteria. This study had a large sample size (*n* = 2,165) and strict exclusion criteria (e.g., secondary craniosynostosis), minimizing random and sampling errors, thus better reflecting the true anomaly detection rate.

This study found that the detection rate of postural cranial abnormality in preterm infants correlates with corrected age. The overall abnormality rate, as well as the detection rates of Plagiocephaly, Brachycephaly, and Asymmetrical Brachycephaly, all exhibit a “U” shape curve pattern. Compared to the 0–2 months group, the detection rate decreased in the 3–4 months group but increased again in the 5–6 months group. This pattern differs from previous preterm studies. Sascha Ifflaender et al. (17) reported anomaly detection rates of 34% at discharge, 46% at 3 months corrected age, and 27% at 6 months corrected age. The high initial rate in the 0–2 months group may be attributed to the extreme immaturity of the preterm skull, prolonged supine positioning and immobilization, weak neck muscles limiting spontaneous head turning, and potential pressure from oxygen therapy devices. Some deformities spontaneously resolve between 3–4 months corrected age due to neurological development, improved positioning management, and natural skull growth. Deformities persisting or progressing by 5–6 months corrected age, coupled with increased caregiver awareness leading to more consultations for suspected abnormalities, likely contribute to the observed increase in detection rate.

Detection rates for different abnormality types varied across age groups. Consistent with international other research centers findings of plagiocephaly being most common (18), plagiocephaly ranked first across all corrected age groups in this study. This is likely due to prolonged supine sleeping positions and positional preferences in infancy. Scaphocephaly was second to plagiocephaly in the 0–2 months and 3–4 months groups but decreased significantly in the 5–6 months group. At 5–6 months, the detection rate of brachycephaly was significantly higher than in the 0–2 months group (19.8% vs 12.7%). This increase might be linked to caregivers adjusting sleep positions, potentially leading to prolonged supine positioning in early development, causing occipital flattening and reduced anteroposterior diameter.

Based on cranial data from 2,165 preterm infants, this study preliminarily proposes diagnostic criteria for postural cranial abnormality in this population, though their applicability requires further validation. Referencing previous research using the ≥75th percentile as abnormal (15), this study provides the first percentile reference values for cranial parameters in preterm infants, offering a revised version of existing international diagnostic standards. The *P*_75_ for CVA in this study was 6.30, close to the lower limit of the international plagiocephaly standard (CVA ≥ 6 mm). The *P*_75_ for CVAI was 4.50%, slightly higher than the international standard of 3.5% (13). The proportion of infants meeting CVAI ≥ 3.5% was much higher than those meeting CVAI ≥ 4.5%. The P_75_ for CR was 0.86, lower than the international brachycephaly standard of 0.90. Using CR ≥ 0.90, 20% brachycephaly was detected; using *P*_75_ (CR = 0.86) as a potential warning threshold would significantly increase this proportion. This strongly advocates for establishing localized diagnostic thresholds based on large 3D scan datasets of Chinese preterm infants, stratified by gestational age and corrected age, rather than simply applying international standards or term infant data. The high anomaly detection rate of scaphocephaly (CR < 0.75, 14.0%) also warrants further investigation into its clinical significance.

Limitations include the single-center cross-sectional design, but the results offer clear implications for clinical practice. Future efforts should focus on early screening for postural cranial abnormality, especially for infants discharged from the NICU. For preterm infants aged 0–2 months corrected age, proactive positional management (e.g., alternating supine and side-lying positions) and physical therapy should be implemented to improve plagiocephaly, utilizing the window of natural improvement and peak cranial plasticity. For infants aged 5–6 months corrected age, orthotic helmet therapy should be actively considered to prevent progression. Future multicenter studies are urgently needed to establish diagnostic thresholds and risk prediction models for cranial parameters in Chinese preterm infants to optimize prevention and treatment systems. This study excluded a small number (0.55%) of infants who received cranial orthotic treatment. Although sensitivity analysis indicated that this exclusion did not significantly influence the results, a weak selection bias cannot be completely ruled out. Future prospective studies should include all consecutive cases and document the indications for intervention to further validate our findings. Furthermore, the existing diagnostic criteria for postural cranial abnormality are all formulated for full-term infants. Although in this study we used corrected gestational age to reduce diagnostic errors, considering that the cranial bone maturity and growth patterns of premature infants differ from those of full-term infants, their applicability still needs to be evaluated. Therefore, in the future, it is urgently necessary to establish diagnostic thresholds that are age-specific and corrected gestational age-specific for premature infants. In addition, it should be noted that we did not collect data on the reason for the first visit (e.g., head shape concern vs. routine follow-up). This limits our ability to assess the extent to which referral or selection bias may have influenced the observed prevalence rates. If a substantial proportion of visits were prompted by parental concerns about head shape, the detection rate may be overestimated. Conversely, if most visits were routine follow-ups, the estimates may more closely reflect the general preterm population. Future studies should record the reason for visit to better understand the impact of healthcare-seeking behavior on prevalence estimates.

## 5 Conclusion

Utilizing 3D scanning technology, this study reveals a high incidence of postural cranial abnormality in Chinese preterm infants, predominantly plagiocephaly. The incidence of postural cranial abnormality in premature infants shows a U-shaped distribution with respect to the corrected gestational age, highlighting the necessity for early and sustained intervention. Furthermore, existing international diagnostic criteria may not be fully suitable for Chinese preterm infants, underscoring the urgent need for localized standards. This study provides evidence to support incorporating cranial screening into routine follow-up for preterm infants and implementing early intervention strategies. The subjects of this study all come from a single hospital, which may introduce selection bias, thereby limiting the generalizability of the conclusions. Future research should focus on multi-center collaboration, large sample sizes, and cross-sectional surveys encompassing multiple factors. It is important to note that this study is a cross-sectional survey, and the observed age group patterns should not be interpreted as individual longitudinal development trajectories.

## Data Availability

The original contributions presented in the study are included in the article/Supplementary Material, further inquiries can be directed to the corresponding author.
